# Discordances between follicle stimulating hormone (FSH) and anti-Müllerian hormone (AMH) in female infertility

**DOI:** 10.1186/1477-7827-8-64

**Published:** 2010-06-17

**Authors:** Norbert Gleicher, Andrea Weghofer, David H Barad

**Affiliations:** 1 The Center for Human Reproduction (CHR) - New York and Foundation for Reproductive Medicine, New York, NY, USA; 2 Department of Obstetrics, Gynecology and Reproductive Sciences, Yale University School of Medicine, New Haven, CT, USA; 3 Department of Obstetrics and Gynecology, Vienna University School of Medicine, Vienna, Austria; 4 Department of Epidemiology and Social Medicine and Obstetrics, Gynecology and Women's Health, Albert Einstein College of Medicine, Bronx, NY, USA

## Abstract

**Background:**

Follicle stimulating hormone (FSH) and anti-Müllerian hormone (AMH) represent the two most frequently utilized laboratory tests in determining ovarian reserve (OR). This study determined the clinical significance of their concordance and discordance in female infertility patients.

**Methods:**

We investigated 366 consecutive infertility patients (350 reached IVF), excluding women with polycystic ovarian syndrome (PCOS). They were considered to have normal FSH and AMH if values fell within age-specific (as-) 95% confidence intervals (CI), and to suffer from diminished ovarian reserve (DOR) if FSH exceeded and/or AMH fell below those. The two hormones, thus, could be concordant (Group I), both normal (IA) or abnormal (IB), show normal AMH/abnormal FSH (Group II) or normal FSH/abnormal AMH (Group III). Oocyte yields, stratified for age categories, were then studied in each group as reflection of OR.

**Results:**

Oocyte yields significantly decreased from groups IA to II to III and IB. Predictive values of as-FSH/AMH patterns changed, however, at different ages. Except at very young and very old ages, normal as-AMH better predicted higher oocytes yields than normal as-FSH, though above age 42 years normal as-FSH predicts good oocyte yields even with abnormally low AMH. Under age 42 discrepancies between as- FSH and as-AMH remain similarly predictive of oocyte yields at all ages.

**Discussion:**

Concordances and discordances between as-FSH and as-AMH improve OR assessments and predictability of oocyte yields in IVF.

## B**ackground**

A patient's ovarian reserve (OR) determines prognostic chances of fertility treatments [1,2] and her treatment options [3-5]. Best possible assessments of OR, therefore, represent a core issue in modern infertility care. Various methodologies have been applied to maximize accuracy of OR determinations, though none has been universally accepted as superior to others [1,6,7]. Follicle stimulating hormone (FSH) still represents the most widely utilized tool in routine daily practice [8,9], though antral follicle counts and anti-Müllerian hormone (AMH) have increasingly attracted proponents [8-12].

We in recent years proposed, independent of OR tests utilized, that normal cut off values be defined in age-specific (as-) ways [13,14]. Since OR declines with advancing female age [2], and "normal", therefore, changes, normal ranges should change in parallel with naturally rising FSH, declining AMH and decreasing antral follicle counts. Yet, OR tests only rarely are utilized in as-ways, diminishing their sensitivity and specificity.

**Figure 1:**
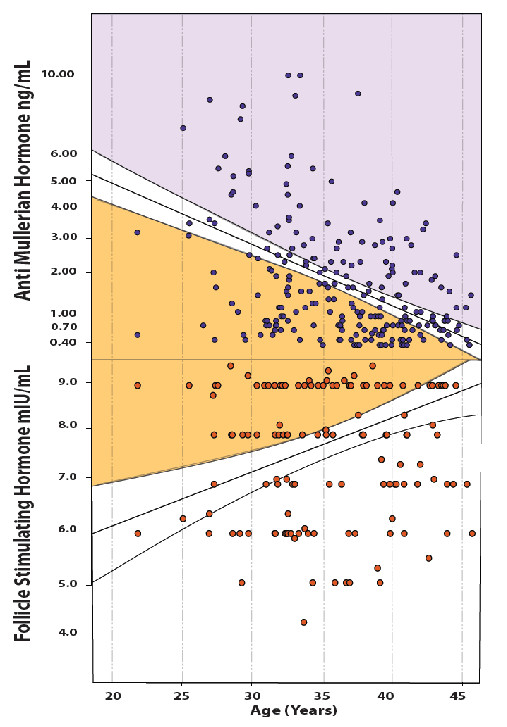
Individual FSH and AMH levels against nomogram of as- levels*. * Some scatters fell outside the shown nomogram field and, therefore, are not shown. as- FSH and AMH levels, represented by the nomogram were established based on 95% CI of FSH and AMH levels of an infertile patient population [30].

Based on 95% confidence intervals (CI) at various ages, we in our center's patient population established normal as-cut off values for FSH [13] and AMH [14] (Figure 1), demonstrating superiority of as-values over standard cut offs in defining oocytes yields with in vitro fertilization (IVF). Since oocytes yields, ultimately, represent the most accurate reflection of OR, these observations support utilization of as-cut offs over non-age specific values.

FSH and AMH, in principle, correlate and cross-corollaries can, indeed, be established [8]. They, however, do not measure identical OR parameters. FSH mostly reflects the last two weeks of follicular maturation when follicles become gonadotropin sensitive, while AMH is mostly representative of the young, post-primordial to preantral follicle pool going through earlier stages of folliculogenesis [15-17].

These differences are reflected in how both hormones are utilized in fertility therapy. For example, a number of studies recently suggested that AMH, better than FSH, reflects in vitro fertilization (IVF) outcomes, including pregnancy chances [9,18]. Figure 1, reflecting as-FSH and AMH levels, also demonstrates this fact by showing at all ages narrower normal ranges for AMH than FSH and, therefore, better specificity.

Utilizing a nomogram for as-AMH (Figure 1, upper panel) and as-FSH (Figure 1, lower panel), despite general statistical congruity between FSH and AMH [8], many individual patient will be discordant. What such discordances clinically indicate is, however, unknown and was, therefore, subject of this investigation.

## Methods

### Patient selection and stratification

This study investigated 366 consecutively presenting female infertility patients from whom baseline FSH (cycle days 2/3) and random AMH were obtained following initial consultation. Patients with established diagnoses of polycystic ovary syndrome (PCOS) by history (oligoaamenorrhea, hirsutism, obesity), laboratory evaluations (FSH/LH inversion, androgen excess, insulin resistance) or ultrasound (polyfollicular ovaries) were excluded but the study included patients who, either based on laboratory or ultrasound evaluation at our center, were for the first time diagnosed with PCOS.

Our center's patient population at all ages includes a disproportionally high number of women with diminished ovarian reserve (DOR) [13]. It, therefore, does not necessarily reflect typical populations seen in most fertility centers. In documentation, Table 1 summarizes patient characteristics.

**Table 1:** Patient characteristics of patients reaching IVF, based on concordance or discordance between as-FSH and as-AMH

	IA	II	III	IB
**N**	48	115	19	168
**Age **(years)*	36.7 ± 4.8	37.5 ± 5.3	36.2 ± 6.6	38.3 ± 5.2
**FSH **(mIU/mL)**	6.5 ± 1.1	15.1 ± 12.8	6.6 ±. 9.0	23.0 ± 21.3
**AMH **(ng/mL)**	3.42 ± 0.8	2.7 ± 3.0	0.6 ± 0.5	0.5 ± 0.4
**Oocytes **(n)**	13.1 ± 7.6	10.9 ± 7.3	9.1 ± 8.2	5.9 ± 5.2
**Race: n **(%)				
*African America*	10(18.5%)	16(29.6%)	2(3.7%)	26(48.1%)
*Asian*	11(19.6%)	21(37.5%)	3(5.4%)	21(37.5%)
*Caucasian*	25(11.5%)	72(33.0%)	11(5.0%)	110(50.5%)
*Middle Eastern*	2(9.1%)	6(27.3%)	3(13.6%)	11(50.0%)
Primary Infertility Diagnoses: n (%)***				
*DOR*	14(9.0%)	44(28.2%)	6(3.8%)	92(59.0%)
*Endometriosis*	2(10.5%)	5(26.3%)	1(5.3%)	11(57.9%)
*Male*	12(12.1%)	37(37.4%)	7(7.1%)	43(43.4%)
*Other*	2(10.5%)	9(47.4%)	2(10.5%)	6(31.6%)
*PCO*	8(36.4%)	9(40.9%)	(0.0%)	5(22.7%)
*Tubal*	10(30.3%)	10(30.3%)	3(9.1%)	10(30.3%)
*Uterine*	0(0.0%)	1(50.0%)	0(0.0%)	1(50.0%)

* Age does not significantly differ between groups;

**FSH, however, progressively increases (p < 0.001) and AMH and oocytes progressively decreased across categories from groups IA, to II, III and IB (p < 0.001).

*** Percentages add up to 100% within each primary diagnosis. By far the most frequently represented primary diagnosis (156/350 patients, 44.6%) was DOR. Amongst those patients Group IB was significantly more prevalent than the three other study groups (p < 0.01).

Amongst 366 consecutive patients, 350 had reached a first IVF cycle for which cycle outcomes, including oocytes yields, were available by time of this data assessment. Patients were stratified into the following age categories: < 34.0; 34.0-35.9; 36.0-37.9; 38.0-39.9; 40.0-41.9; and ≥ 42 years. Within each age category, they were considered to have age-appropriate or abnormal as-FSH and AMH levels. Normal as-levels for FSH and AMH for our patient population have previously been reported [13,14] and are demonstrated graphically in Figure 1.

Patients were then assessed in four groups: Group [IA], as-FSH and -AMH were both in normal range; [II], as-AMH normal and as-FSH abnormal (high); [III], as-AMH abnormal (low) and as-FSH normal and, finally, [IB], both at abnormal as-ranges (FSH high and AMH low). Oocyte yields and other IVF cycle outcome parameters were recorded in first IVF cycles.

### DHEA supplementation and ovarian stimulation

Since our center routinely supplements DOR patients with dehydroepiandrosterone (DHEA) prior to IVF cycles [19], the high prevalence of DOR in our patient population (Table 1) resulted in supplementation to patients at all ages in Groups IB, II and III and also to women above age 38 years in Group IA. Following at least six weeks of DHEA supplementation, patients are then stimulated in their first cycle with a microdose agonist cycle with FSH preponderance (at least 300 IU) and human menopausal gonadotropin (hMG, 150 IU) [20]. This means that all patients in Groups IB, II and III and women above age 38 years in Group IA received such high dose gonadotropins stimulation. Group IA patients under age 38 years uniformly received ovarian stimulation in a long agonist protocol with maximally 300 IU of gonadotropins, usually hMG rarely split between hMG and FSH. IVF is performed in routine fashion and embryos are transferred on day-3 after retrieval.

Estradiol, FSH and AMH were assayed in house, as previously reported {Estradiol and FSH [21]; AMH [22]}, utilizing standard ELISA assays (AIA-600 II, Tohso, Tokyo, Japan and Diagnostic Systems Laboratories, Inc. Webster, TX 77598-4217, USA, respectively).

### Statistics

Normally distributed data were compared by means of one-way analysis of variance test. All data are expressed as mean ± standard deviation; a p-value < 0.05 was considered statistically significant. All analyses were carried out with SPSS software for Windows version 17.0, 2005 (SPSS Inc. Chicago, IL)

### Institutional Review Board

Patients, at time of initial consultation, sign an informed consent form, allowing review of medical records for research purposes as long as patient identity remains protected and records remain anonymous. Studies involving record review, and meeting these criteria, therefore, undergo expedited review by the center's Institutional Review Board (IRB). Confirmation from the center's IRB chairman is available upon request. The center also, in addition, maintains a protected electronic research data collection system with controlled access, which guarantees that above noted conditions are met.

## Results and Discussion

Mean age for these 350 women was 37 ± 5.6 years; mean FSH was 17.0 ± 0.93 mIU/mL, while mean AMH was 1.59 ± 0.12 ng/mL. Table 2 offers mean FSH and AMH levels for the six age categories.

**Table 2:** Age-stratified as-AMH and as-FSH levels in all four study groups

Age (years)	Group	N	AMH (ng/mL	FSH (mIU/mL)
**≤ 34.00**	**IA**	17	5.1 ± 2.6	6.1 ± 0.5
	**II**	31	4.0 ± 2.0	14.5 ± 17.3
	**III**	10	0.9 ± 0.5	6.3 ± 0.9
	**IB**	35	0.9 ± 0.5	20.4 ± 15.9
**34.01 - 36.00**	**IA**	4	2.2 ± 0.6	6.0 ± 0.8
	**II**	16	4.3 ± 6.7	11.6 ± 7.5
	**III**	-	-	-
	**IB**	11	0.4 ± 0.4	16.8 ± 10.9
**36.01 - 38.00**	**IA**	5	2.6 ± 0.7	6.3 ± 1.5
	**II**	8	3.3 ± 2.4	11.2 ± 3.5
	**III**	1	0.6	6.0
	**IB**	25	0.7 ± 0.4	18.0 ± 15.0
**38.01 - 40.00**	**IA**	7	4.4 ± 4.9	6.9 ± 1.3
	**II**	15	1.9 ± 1.0	13.9 ± 7.1
	**III**	2	0.3 ± 0.1	7.8 ± 0.4
	**IB**	29	0.5 ± 0.3	22.7 ± 20.8
**40.01 - 42.00**	**IA**	10	1.8 ± 1.4	6.9 ± 1.5
	**II**	17	1.4 ± 1.0	16.7 ± 12.0
	**III**	1	0.3	6.3
	**IB**	24	0.3 ± 0.2	23.9 ± 17.1
**> 42.00**	**IA**	5	1.7 ± 1.2	7.0 ± 1.0
	**II**	28	1.3 ± 0.7	17.8 ± 13.2
	**III**	5	0.2 ± 0.2	6.8 ± 0.8
	**IB**	44	0.2 ± 0.2	20.1 ± 14.3
**Total means**		350	1.6 ± 2.4	16.1 ± 14.3

Figure 1 demonstrates individual FSH and AMH values against a previously established nomogram of as-levels. Since PCOS patients were excluded, and since the purpose of this study was definition of DOR, only lower cut off values of AMH are relevant.

Amongst 350 IVF patients 229 (65.8%) were concordant in FSH and AMH in that they were either age-specifically normal or abnormal in both (Group I). Fifty (14.4%) showed normal as- values (Group IA) and 166 (47.7%) abnormal values in both hormone (Group 1B); 115 (33.1%) were discordant in that as-AMH was normal and as-FSH was abnormally high (Group II); and 19 patients (5.5%) were discordant with abnormally low as-AMH and normal as-FSH (Group III). Table 1 summarizes their characteristics.

As Table 1 demonstrates, ages did not differ between groups. FSH levels, however, progressively increased (F = 17.7, df = 3, p < 0.001 and AMH (F = 40, df = 3, p < 0.001) as well as oocyte yields (F = 22.2, df = 3, p < 0.001) progressively decreased from Group IA to Group II and Group III and, finally, Group IB.

**Figure 2:**
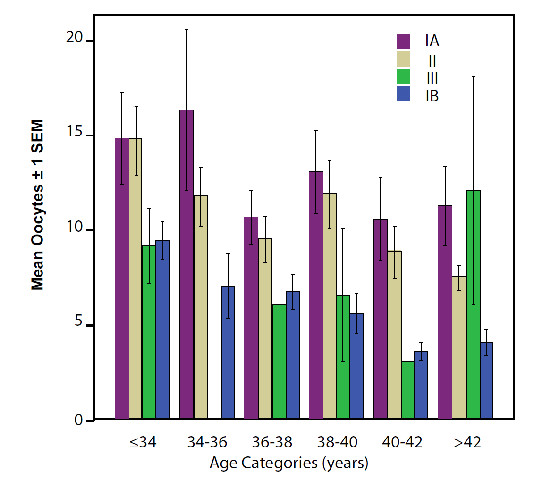
as-Oocyte yield, based on concordance or discordance of as-FSH and as-AMH*. * Groups are color-coded in accordance with legend in figure.

Figure 2 translates these findings into an assessment of OR based on oocyte yields. As the figure demonstrates, oocyte yields were at all ages uniformly the highest in Group IA. At youngest ages (≤ 34 years) Groups IA and II produce practically identical oocyte yields, as do Groups III and IB, though the latter two at significantly reduced numbers (Groups IA, II: 15.0 ± 9.9, n = 48; Groups III, IB, 9.3 ± 5.9 N = 45, P = 0.001). At age categories 34-36 through 42, oocyte yields appear to follow the general OR trend, outlined in Table 2, from Group IA to Group II, to Group III and Group IB. At older age (>42), oocyte yields, however, become more erratic, with Groups IA and III demonstrating similar and significantly higher oocyte yields than Groups II and IB. (Group 1A, III: 11.6 ± 9.5, n = 10; Group III, 1B: 5.4 ± 4.4, p = 0.001).

Correct definition of OR is a corner stone of infertility therapy. It, therefore, is not surprising that investigators are attempting to improve OR assessments. Amongst methods currently utilized, FSH and AMH appear to have achieved primacy [6,7,9,12-16,18]. Both define OR reasonably well and, in principle, correlate [8]. They, however, do, as this study suggests, diverge in 38.3 percent of cases (Table 1)

We [9,14] and others [18] demonstrated that AMH offers better clinical specificity than FSH in regards to IVF outcome parameters, including establishment of pregnancy. This is also suggested by the fact that normal as-AMH levels, as shown in Figure 1, at all ages are in a narrower range than is as-FSH.

Better specificity for AMH should not surprise because AMH reflects the post-primordial and pre-antral follicle population, while FSH is mostly reflective of late-stage follicles in the gonadotropin-sensitive stage, preceding ovulation by only approximately two weeks [15-17]. AMH, therefore, better reflects the follicle "pool," though an ideal measurement of OR should, likely, also include the still unrecruited primordial follicle pool.

These follicle populations change with advancing female age. Follicle recruitment declines and follicles reaching gonadotropin sensitivity also decline in numbers [23] One, therefore, can expect that FSH and AMH may differently reflect OR at different ages.

This hypothesis was, indeed, confirmed in this study, which investigated the meaning of concordance and discordance between as- FSH and as-AMH levels at varying ages. We chose to investigate as-hormone, rather than non-age specific, levels because we have previously demonstrated that as-OR testing with FSH and AMH is superior in predicting oocyte yields to non-age-specific testing [13,14].

Table 1 demonstrates that, combined for all study subjects, FSH increases and AMH as well as oocyte yields decline from Group IA (both hormones in normal as-range) to Group II (AMH normal, FSH elevated), to Group III (FSH normal, AMH low) to Group 1B (FSH abnormally high, AMH abnormally low).

This observation suggests that, in principle, normal AMH is more important than normal FSH. Figure 2, however, demonstrates a more complicated picture when data are analyzed based on age categories. The figure shows that in the youngest patient group (≤ 34 years), based on oocyte yields, there is really no difference in OR between women in Groups IA and II, just as there is no difference in oocyte yields between Groups III and IB, with the latter two groups, both, significantly lower than the first two. Young infertility patients with DOR, therefore, will still have excellent oocyte yields whether AMH and FSH or whether only AMH is in normal range. They will, however, have clearly reduced oocyte yields if only FSH is in normal range or both hormones are abnormal.

This finding correlates with the previously reported observation that elevated FSH in younger females is much less ominous than in older patients [24,25] but suggests that this observation only applies if AMH is still in normal range. These observations, thus, once more, demonstrate superiority of AMH over FSH in reflecting OR in younger patients.

At age 34, this pattern, however, starts changing: Between ages 34 and 42 years, oocyte yields follow the declining oocyte yield pattern of the whole study group, noted in Table 1, with largest egg numbers in Group IA, declining in Group II, further declining in Group III and, ultimately, being the lowest in Group IB. In this age category being normal in both hormone parameters (Group IA), uniformly, reflects best prognosis for good oocyte yields, followed in order by normal AMH/abnormal FSH (Group II), normal FSH/abnormal AMH (Group III) and both values abnormal (Group IB). At that age range, normal AMH is, therefore, also clearly better than normal FSH in predicting better oocyte yields. Indeed, once AMH levels are abnormal, even if FSH is still normal (Group III), oocyte yields are low in comparison.

Figure 2 also demonstrates that, above age 42, this picture once again, changes. At such advanced age, normal FSH better predicts nominally higher oocyte yields than AMH as Group III (normal FSH, abnormal AMH), produces surprisingly good oocyte yields, - indeed, similar to those of Group IA (though these trends do not achieve statistical significance). Why normal FSH at such advanced female age would have such a positive predictive value in comparison to AMH remains to be determined and is currently still under investigation. It is tempting to speculate that normal FSH at this age is (via negative feedback) reflective of normal follicular estradiol production, and, therefore, suggestive of better quality follicles. Alternatively, abnormally low AMH could be unable to inhibit follicular recruitment and, the in Group III observe constellation in women above age 42 years could, simply, reflect a subgroup of women who at this advanced age still more actively recruit follicles.

As we already previously reported [26,27], these data, once again, confirm that accurate OR assessments are the most difficult at youngest and oldest ages. This fact is also demonstrated in the widening ranges of normal FSH and AMH values (Figure 1) at both age extremes. Why that should be is, once again, speculative but it seems likely that these wider ranges of normal, simply, are reflective of greater heterogeneity of ovarian function at very young and old ages.

Figure 1, however, also demonstrates that normal as-ranges for FSH and AMH are the narrowest at approximately ages 33 to 34 years. As noted before, this is exactly the age break point in this study between younger women with the typical FSH/AMH pattern of younger age and the vast majority of middle aged patients with the previously described declining oocyte yield pattern from Groups IA to II, III and IB. Interestingly, this is also the age, where the OR of women with normal CGG counts on the *FMR1 *(fragile X) gene falls below the OR of heterozygous-abnormal women [28].

Combined, these last two observations suggest that heterogeneity of OR at very young and old ages may be genetically predetermined. As OR declines with advancing age, heterogeneity is lost and the age of approximately 34 years appears to represents an important switching point in human OR.

Figure 2 also allows for additional observations: When Group I patients are followed longitudinally from youngest to oldest ages, mean oocyte yields decrease remarkably little. Indeed, even women above age 42 still produce an average of over 10 oocytes per cycle. This may surprise, considering that most here investigated patients were afflicted by DOR (Table 1), usually characterized by ovarian resistance to stimulation with gonadotropins and poor oocyte yields [29]. Indeed, some investigators equate these two conditions and the literature, therefore, often defines DOR as retrieval of four or less oocytes [30].

Such definitions, however, for a number of reasons, do not apply here: First, the here utilized definition of DOR relies on as- FSH and AMH levels, which previously have been demonstrated to differentiate between significantly lower and higher oocyte yields [13,14]. Second, most here investigated patients, because of DOR diagnoses, were supplemented with dehydroepinadrosterone (DHEA), which has been reported to improve oocyte yields significantly [19]. Finally, because of DOR, most patients in this study received maximal ovarian stimulation with at least 450 IU of gonadotropins in a routine microdose agonist cycle [20].

In short, as-OR evaluations offered more sensitive DOR detection prior to stimulation, while DHEA and appropriately chosen ovarian stimulation protocols enhanced oocyte yields. Better than expected oocyte yields in this patient population should, therefore, not surprise, though even we were surprised by how excellent yields were in some women of very advanced ages (Figure 2).

As expected, all four patient groups demonstrated decreasing oocyte yields with advancing age. They uniformly, however, were small: Groups IA and II, with best oocyte yields at very young ages, maintain their numerical advantage up to age 42 years. Similarly, Groups III and IB, at young ages demonstrating significantly reduced oocyte yields, remain at retrieval numbers significantly below those of the other two groups until age 42. Only above age 42, Group III suddenly breaks out with surprisingly excellent oocyte numbers.

These slow and persistent decreases in oocyte yields with age in each of the four groups suggest a genetically preprogrammed OR pattern, observable from early age on. The data, therefore, resemble OR patterns we described in infertility patients, based on normal, heterozygous or homozygous abnormal *FMR1 *status [28]. We, therefore, are currently investigating whether the patients' here observed FSH/AMH patterns correlate to their *FMR1 *status.

## Conclusions

This study defines four specific FSH/AMH patterns and their effects on oocyte yields at different ages. While women with normal as-FSH and normal as-AMH at all ages produce the best oocyte yields, women with normal as-AMH but abnormal as-FSH do not follow far behind until age 42. In contrast to these two groups, women with normal as-FSH but abnormal as-AMH and those with abnormal as-FSH and -AMH at all ages, up to 42 years, demonstrate significantly reduced oocyte yields. After age 42, there is little difference between women with normal FSH and AMH and with either one at abnormal levels. Only women with both hormones at abnormal levels produce reduced egg numbers.

The prospective assessment of as-FSH and -AMH, in combination with female age, thus, can help in predicting oocyte yields in women with infertility. Here presented data should, however, be viewed with caution since studied patients were almost uniformly affected by DOR and, therefore, under DHEA supplementation. Whether these data are also applicable to unsupplemented infertility patients remains to be established.

## Abbreviations

as-: age-specific; AMH: Anti-Müllerian hormone; CI: Confidence interval; DOR: Diminished ovarian reserve; DHEA: Dehydroepiandrosterone; ELISA: Enzyme-linked immunosorbent assay; FSH: Follicle-stimulating hormone; IRB: Institutional Review Board; OR: Ovarian reserve; PCOS: Polycystic ovarian syndrome;

## Competing interests

NG and DHB are co-owners of a recently granted U.S. patent, which claims clinical benefits from DHEA supplementation of women with DOR. Other DHEA patents have been applied for and are pending. Both investigators are also listed as co-inventors on pending patent applications, which, amongst other claims, suggest benefits in the diagnosis of premature DOR from determining number of CGG repeats on the *FMR1 *gene. Other pending patent applications, involving the authors are unrelated to matters discussed in this manuscript. Both authors have received research support, lecture fees and travel reimbursements from various pharmaceutical companies, though none related to the materials presented in this manuscript.

## Authors' contributions

NG and DHB contributed equally to the manuscript. Both participated in design of study and data analysis. DHB performed a majority of the statistical analysis, while NG drafted most of the manuscript. AW participated in study design, data analysis and reviewed the manuscript. All authors read and approved the final manuscript.
